# Actomyosin dynamics in detached cells: linking clutch model to cell migration and cytokinesis

**DOI:** 10.1186/s12860-025-00563-7

**Published:** 2025-12-24

**Authors:** Shigehiko Yumura, Yumi Kikuta, Go Itoh

**Affiliations:** 1 Graduate School of Sciences and Technology for Innovation, Yamaguchi University, Yamaguchi, 753-8512 Japan; 2 Department of Molecular Medicine and Biochemistry, Akita University Graduate School of Medicine, Akita, 010-8543 Japan

**Keywords:** Actin, Cell migration, Cortical flow, Cytokinesis, Myosin, Retrograde flow

## Abstract

**Background:**

Cortical retrograde flow of actomyosin plays a critical role in cell migration and cytokinesis. While such flows have been extensively studied in adherent cells, their underlying mechanisms in non-adherent conditions remain poorly understood. In this study, we investigated actomyosin dynamics in *Dictyostelium* cells detached from the substratum, with a focus on cytoskeletal turnover and the applicability of the clutch hypothesis.

**Results:**

Detached cells exhibited robust retrograde flows of actin and myosin II filaments within the cell cortex, moving at identical velocities. In contrast, membrane components and particles attached to the cell surface showed no movement, suggesting that the flows are driven by cortical actomyosin rather than membrane trafficking. Inhibition of actin polymerization completely abolished the flows, while myosin II inhibition had only a minor effect, indicating that actin polymerization is the primary driver. Flow velocities gradually decreased from the anterior to the posterior, consistent with a model in which actin filaments are pushed rearward rather than pulled from the back. Flow velocity was inversely proportional to cell migration speed, and nascent adhesion foci also moved retrogradely, supporting the clutch hypothesis, although *Dictyostelium* cells have neither canonical integrins nor well-defined extracellular matrix. Photobleaching experiments revealed that actin and myosin II filaments undergo turnover and recycling near the posterior. During cytokinesis under non-adherent conditions, cortical flows from the poles to the equator were significantly enhanced, and membrane components remained static. The clutch hypothesis may explain also the cortical flow and contractile ring formation during cytokinesis.

**Conclusions:**

Our findings demonstrate that detachment from the substratum induces dynamic, turnover-dependent actomyosin flows. These flows are primarily driven by actin polymerization and are consistent with the clutch hypothesis, which helps explain both cell migration and contractile ring formation during cytokinesis. This study expands the applicability of the clutch model and highlights the dynamic nature of the cytoskeleton in non-adherent cells.

**Supplementary Information:**

The online version contains supplementary material available at 10.1186/s12860-025-00563-7.

## Background

Amoeboid movement is a fundamental characteristic of various migrating cells, including protozoan amoebae, leukocytes, carcinoma cells, neuronal cells, and *Dictyostelium* cells. This type of movement resembles an inchworm-like process and involves three key steps: extension of a leading pseudopod, adhesion to the substratum, and retraction of the posterior, which propels the cell body forward [1].

The extension of the pseudopod is driven by actin polymerization mediated by the Arp2/3 complex [2]. Cell-substratum adhesion occurs at focal adhesions, which connect the substratum to the intracellular actin cytoskeleton via transmembrane proteins such as integrin. This connection transduces mechanical force to the substratum, generating traction forces. Alternatively, in three-dimensional matrices or confined spaces, cells employ a migration mechanism that relies on friction rather than focal adhesions [3–6].

Myosin II is localized at the rear of migrating cells and plays a crucial role in actively retracting the posterior cortex through actomyosin-driven mechanisms. This retraction also facilitates detachment from the substratum. Actin polymerization at the leading edge is closely linked to forward cell migration, and the retrograde flow of actin exhibits an inverse correlation with cell velocity [7, 8]. The ‘clutch hypothesis’ describes the interplay between focal adhesions, the actin cytoskeleton, and cell migration [9–11]. When the clutch is engaged, the actin cytoskeleton remains coupled to the substratum, facilitating forward movement. In contrast, disengagement of the clutch results in actin polymerization driving retrograde flow, thereby impeding cell advancement [12]. According to the clutch hypothesis, coupling between the cytoskeleton and substratum transmits traction forces [13], which involves multiple points of slippage rather than a rigid connection [14, 15].

While retrograde flow was initially attributed solely to actin polymerization, myosin II may also contribute to its generation [16, 17]. However, the molecular mechanisms underlying the clutch hypothesis remain unclear, and its general applicability across different cell types requires further investigation.

*Dictyostelium* cells have served as a model system for studying cell migration and cytokinesis. Previous studies have demonstrated that cortical actin and myosin II filaments remain stationary relative to the substratum during cell movement. However, when cells detach from the substratum, actin and myosin II filaments exhibit a robust retrograde flow from the anterior to the posterior end [18].

In this study, we establish an inverse relationship between retrograde flow and cell velocity, supporting the applicability of the clutch hypothesis to *Dictyostelium* cell migration, although *Dictyostelium* cells have neither canonical integrins nor well-defined extracellular matrix. Furthermore, we identify actin polymerization at the anterior as the primary driver of retrograde flow. Additionally, we observe the disassembly and recycling of actin and myosin II at the posterior region. In dividing cells, we also note an enhanced cortical flow from the poles to the equator upon detachment. This study expands the applicability of the clutch model.

## Methods

### Cell culture

*Dictyostelium discoideum* (AX2) and mutant cells were cultured in plastic dishes or under shaking conditions (150 rpm) at 22 °C in HL5 medium (1.3% bacteriological peptone, 0.75% yeast extract, 85.5 mM D-glucose, 3.5 mM Na_2_HPO_4_·12H_2_O, and 3.5 mM KH_2_PO_4_, pH 6.3), as previously described [19].

### Plasmids and transformation

Cells were transformed using extrachromosomal vectors for the expression of GFP-lifeact [20], GFP-cAR1 [21, 22], and GFP-myosin II [19] through electroporation or laserporation, as described earlier [23, 24]. Transformed cells were selected in HL5 medium in plastic dishes containing 10 µg/mL G418 (Wako Pure Chemical Corporation, Osaka, Japan).

### Cell preparation for microscopy

To create non-adherent conditions, three methods were employed. Firstly, talin A/B null cells were placed on a glass-bottom chamber and mildly overlaid with a 2 mm-thick agarose block (1.5% agarose solved in the same buffer used for the medium ) to improve cell imaging and stabilize the passive movement of detached cells, as described previously [25, 26]. Secondly, cells were detached in a EDTA solution (10 mM NaCl, 10 mM KCl, 2 mM EDTA, 0.1 mM MgCl_2_, and 2 mM MES, pH 6.3). After 30 min., all cells were detached from the substratum [18]. The detached cells were settled on a glass bottom dish and overlaid with a 2 mm-thick agarose block containing EDTA solution. Thirdly, cells were placed on a Lipidure-coated glass-bottom chamber and agar-overlaid. The coverslip surface was spin-coated with 0.5% (w/v) Lipidure [6]. Usually, experiments were started 30 min after agar-overlay.

Cells were fixed in cold ethanol containing 1% (v/v) formalin and stained with 100 nM tetramethyl rhodamine-phalloidin [25].

The effects of inhibitors, latrunculin A (Sigma-Aldrich, Japan), jasplakinolide (Sigma-Aldrich, Japan), and blebbistatin (Sigma-Aldrich, Japan), were examined by applying a small aliquot of the solution containing the inhibitor to achieve a final concentration of 1 µM, 4 µM, and 150 µM, respectively. To observe particles attached to the cell surface, cells were placed in a glass-bottomed chamber. A small amount of medium containing carboxylated beads (0.5 μm in diameter, Molecular Probes) was applied to the cells using a micropipette, as previously described [18].

### Microscopy

Fluorescence and phase-contrast images of cells expressing fluorescent proteins were observed using a confocal microscope (LSM510META, Carl Zeiss, Oberkochen, Germany) equipped with a 63× objective and an argon laser (with standard filter settings for GFP). The optical thickness was set at 1 μm. The exposure time was 100 ms with an interval of 0.2–10 s. Total internal reflection fluorescence (TIRF) microscopy (based on the IX71 microscope; Olympus) was conducted as previously outlined [27].

### Photobleaching

Photobleaching was performed using a confocal microscope with full power of argon laser (488 nm lines, 30 mW), as described previously [19]. Changes in fluorescence intensity in the bleached area were monitored over time after background subtraction using imageJ/Fiji (https://imagej.net/software/fiji). These data were subjected to curve fitting using GraphPad Prism 8 (GraphPad Software Inc., USA). The time course of recovery was fitted to an equation for a single exponential rise to maximum [19].

### Statistical analysis

Statistical analysis was carried out using GraphPad Prism 8. Data were analyzed using one-way ANOVA with Tukey’s multiple comparison test.

## Results

### Surface wave and retrograde flow of actin and myosin II in detached cells

Previous studies have shown that detachment of *Dictyostelium* cells from the substratum induces a robust retrograde flow of actin and myosin II filaments. In parallel, a novel phenomenon termed “surface wave” was identified, characterized by the retrograde movement of convex-concave deformations on the cell surface [18]. In this study, we further investigated the mechanisms underlying these flows.

To detach cells, we employed three methods: (1) using talin A/B double-knockout (talin A/B null) cells, (2) incubating cells in an EDTA-containing buffer, and (3) culturing cells on a Lipidure-coated substratum to prevent adhesion [6, 28]. To prevent cells from floating away, we applied gentle compression with a hydrophobic agarose block. Observations were conducted using cells exhibiting a highly polarized morphology with a single pseudopod. The side containing the pseudopod is referred to as the “anterior,” even in non-migrating cells. To prevent the cells drifting and to visualize the ventral cortex using TIRF microscopy, they were gently compressed with an agarose block. Over time (typically ~ 1 h after agar-overlay), the cells began to migrate despite their inability to adhere to the substratum. In this confined environment, detached or non-adherent cells can migrate via friction without requiring specific adhesions, a phenomenon observed not only in *Dictyostelium* but also in animal cells [5, 6, 29, 30]. Retrograde flows were analyzed before the onset of migration.

**Fig. 1:**
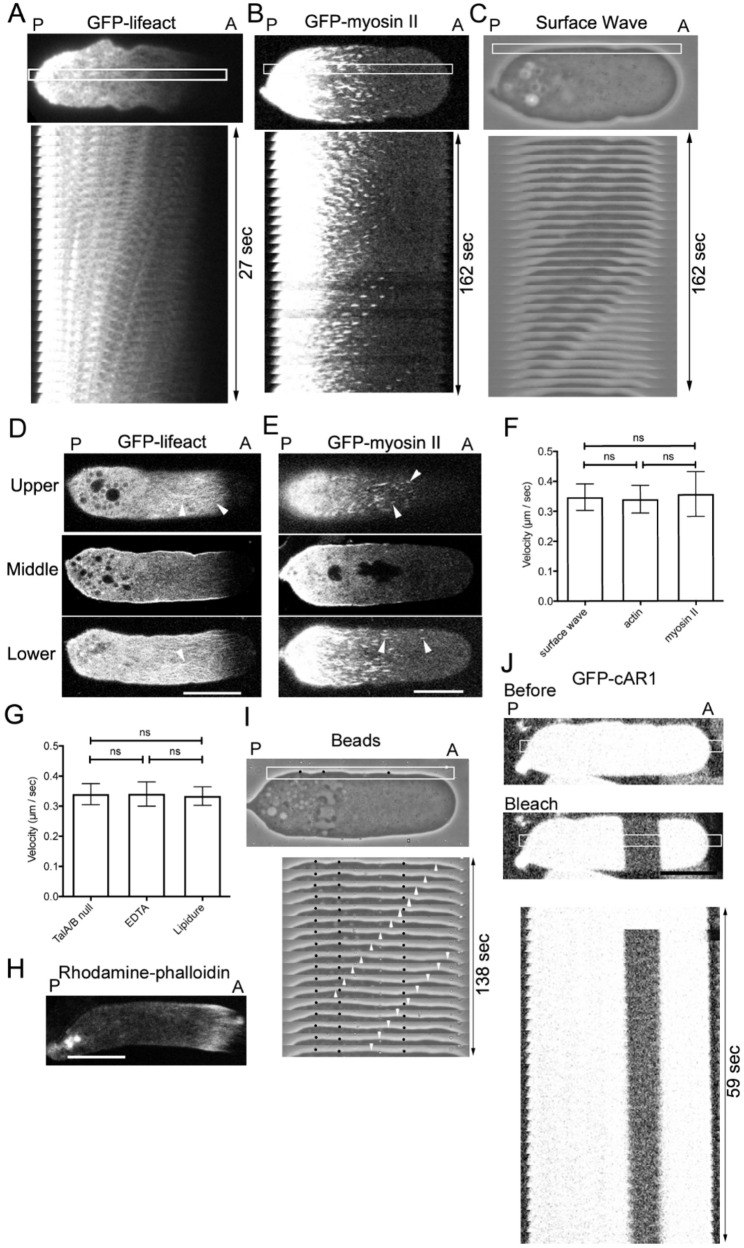
Surface Waves and Retrograde Flow of Actin and Myosin II in Detached Cells (**A-C**) Representative TIRF (**A** and **B**) and bright-field (**C**) images of talin A/B null cells expressing GFP-lifeact (**A**) and GFP-myosin II (**B**) under agar-overlay, respectively. Panels B and C show the same cell. Lower panels display kymograph within the rectangles in the upper panels. Small “A” and “P” indicate anterior and posterior, respectively. (**D**) Three optical sections (upper, middle, and lower) of a cell expressing GFP-lifeact. Arrowheads indicate filamentous structures of actin. (**E**) Three optical sections (upper, middle, and lower) of a cell expressing GFP-myosin II. Arrowheads indicate myosin II filaments. (**F**) Comparison of velocities among surface wave, actin flow, and myosin II flow. Data are presented as mean ± SD (*n* = 6 each) and analyzed by one-way ANOVA with Tukey’s multiple comparison test. ns, not significant, *P* > 0.05. (**G**) Comparison of actin flow velocities among three detaching methods: talin A/B null cells, EDTA, and Lipidure-coated substrate. Data are presented as mean ± SD (*n* = 7, each) and analyzed by one-way ANOVA with Tukey’s multiple comparison test. ns, not significant, *P* > 0.05. (**H**) A typical fluorescence image of a fixed talin A/B null cell stained with rhodamine-phalloidin. (**I**) A typical bright-field image of a talin A/B null cell with small beads (black spots) attached to the cell surface. Lower panels show a kymograph within the white rectangle in the upper panel. Arrowheads shows retrograde flow of surface waves. (**J**) A typical photobleaching experiment of a cell expressing GFP-cAR1. The upper two panels show fluorescence images before and after photobleaching, respectively. The lower panel shows a kymograph within the white box in the upper panel. Panels A-C were obtained by TIRF microscopy and all others were by confocal microscopy. Bars, 10 μm

Representative kymographs of talin A/B null cells depict these flows in Figs. 1A–C. A meshwork of actin filaments (Fig. 1A, Supplementary Movie S1) and individual myosin II filaments exhibited retrograde flow (Fig. 1B, Supplementary Movie S2). These myosin II filaments are bipolar filaments as described previously [27, 31]. The convex-concave deformations of the cell surface, originating near the anterior, propagated backward as surface waves (Fig. 1C, Supplementary Movie S3).

Figure 1D and E show typical optical sections of cells expressing GFP-Lifeact and GFP-myosin II, respectively. Actin and myosin II filament flows were observed in both upper and lower sections, but only at the peripheral cortex in middle sections, suggesting that these flows are confined to the cell cortex. Incidentally, the actin cytoskeleton in *Dictyostelium* cells is primarily localized at the membrane interface, including pseudopods, filopodia, and the actin cortex, while large actin structures such as stress fibers are absent in the cell interior [18, 25].

A comparison of actomyosin flow and surface wave velocities (Fig. 1F) revealed no significant differences (~ 0.35 μm/sec, *n* = 6 for each), implying a shared underlying mechanism. Figure 1G shows no significant difference in actin flow velocities among three detachment conditions, indicating that the flows were a general consequence of detachment.

Notably, the anterior region lacked GFP-Lifeact fluorescence in both live-cell images (Figs. 1A, D). However, rhodamine-phalloidin staining of fixed cells (Fig. 1H) confirmed the presence of actin filaments in this region. When stimulated with a chemoattractant, actin assembly in pseudopods takes 7–10 Sec [32]. The actin flow (approximately 3.5 μm/sec) may be too fast for GFP-Lifeact bind to newly assembled actin filaments at the anterior.

To assess whether the observed surface waves reflect actual membrane flow, we tracked beads adhered to the cell surface. Kymographs (Fig. 1I) showed that while surface waves propagated retrogradely, the beads did not exhibit net displacement (black spots). Photobleaching experiments using GFP-cAR1, a marker for membrane components, further confirmed the absence of retrograde membrane flow (Fig. 1J). These findings suggest that the surface waves resemble the movement of a floating leaf on a waving water surface. These findings suggest that the flow is propelled by the cortical actomyosin rather than the membrane circulation due to the anterior exocytosis and posterior endocytosis [33].

### Actin polymerization primarily drives the flows

**Fig. 2:**
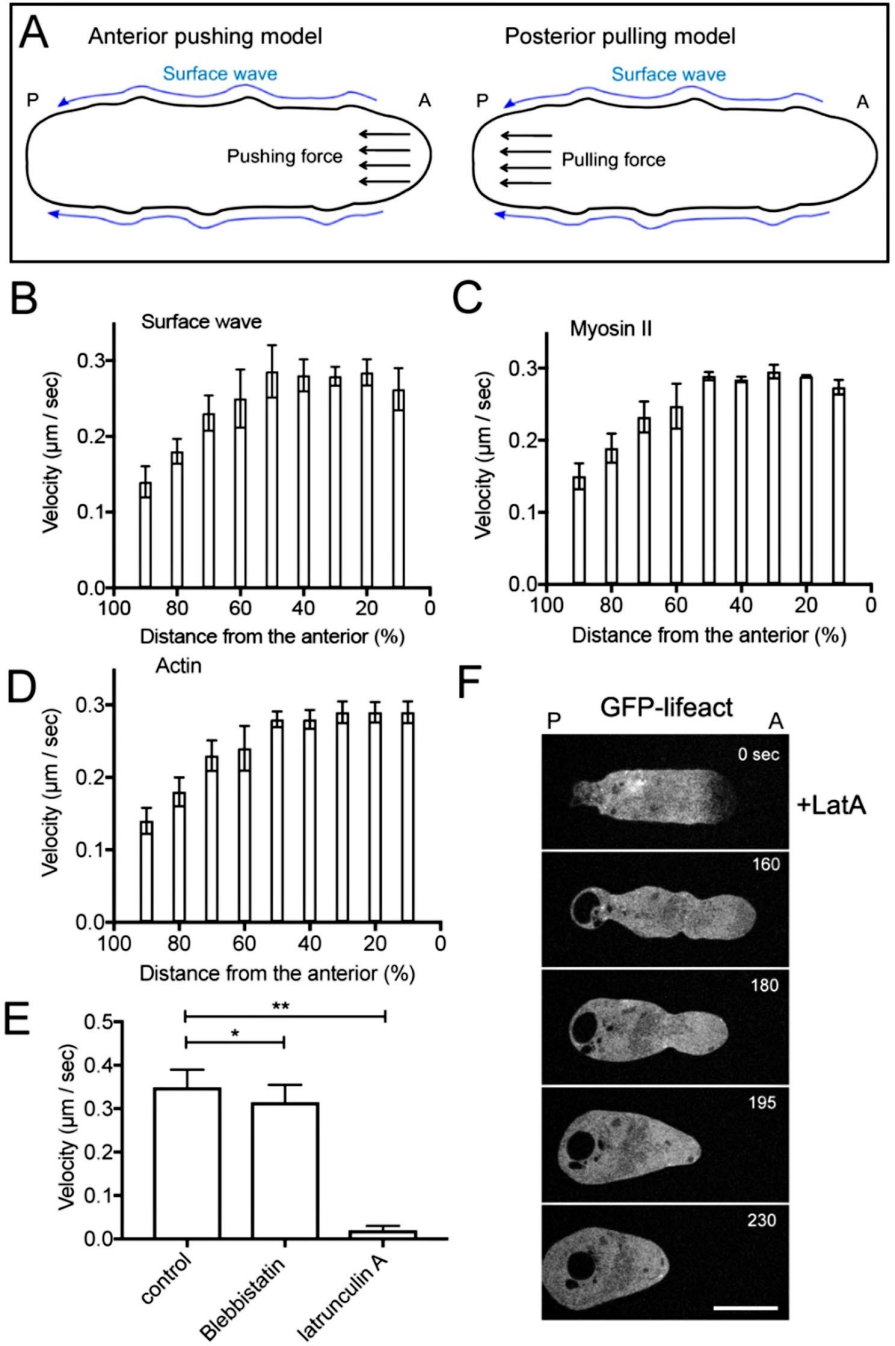
Driving Force of the Retrograde Flows. (**A**) Two models for the driving force of retrograde flows: Anterior pushing model and Posterior pulling model. Small “A” and “P” indicate anterior and posterior, respectively. (**B-D**) Velocities of the surface wave (**B**), myosin II flow (**C**), and actin flow (**D**) along the cell length. Data were derived from multiple cells (*n* = 11 each). The position was normalized to the cell length by the distance from the anterior (%). (E) Comparison of flow velocities of myosin filaments among control, blebbistatin-treated, and latrunculin A-treated cells (*n* = 10 for each). Blebbistatin-treated cells were observed 1 h after treatment, whereas latrunculin-treated cells were observed immediately after treatment. Data are presented as mean ± SD and analyzed by one-way ANOVA with Tukey’s multiple comparison test. ** *p* < 0.0001, ns, not significant, *P* > 0.05. (**F**) A typical confocal image sequence of a cell expressing GFP-lifeact after treatment with latrunculin A (LatA). Similar observations were confirmed in more than 10 cells. Bars, 10 μm

To elucidate the driving force behind these flows, we considered two models (Fig. 2A).

The first model aligns with the clutch model: actin polymerization at the anterior membrane exerts pushing force to extend pseudopods. When cells adhere to a substratum, this force is transmitted forward via the transmembrane proteins such as integrins (clutched). Upon detachment, the absence of a substratum prevents forward transmission, resulting in actin retrograde flow (Anterior pushing model). The second model posits that myosin II, which predominantly localize at the posterior, generates contractile force, pulling actin filaments rearward (Posterior pulling model) [25].

Velocity analyses of the surface wave, actin flow, and myosin II flow revealed a significant deceleration beginning approximately 60% of the cell length from the anterior (Figs. 2B-D). This posterior velocity decrease contradicts the Posterior Pulling Model, supporting the Anterior Pushing Model as the more plausible mechanism.

To further validate this model, we treated cells with blebbistatin, a myosin II ATPase inhibitor. The velocities of myosin II filaments and the surface flows were slightly but significantly reduced (10.5 ± 3.5% decrease, *n* = 20) (Fig. 2E). However, blebbistatin had little effect on overall cell morphology.

In contrast, latrunculin A, an actin depolymerizer, completely halted the flows of actin filaments and surface waves and caused cells to adopt a more rounded morphology due to anterior retraction (Figs. 2E, F, see also Supplementary movie Movie S4).

These findings indicate that actin polymerization is the primary driving force of actomyosin flow and surface waves, with myosin II playing only a minor contributory role.

### Velocity of the flows is inversely proportional to cell migration velocity

**Fig. 3:**
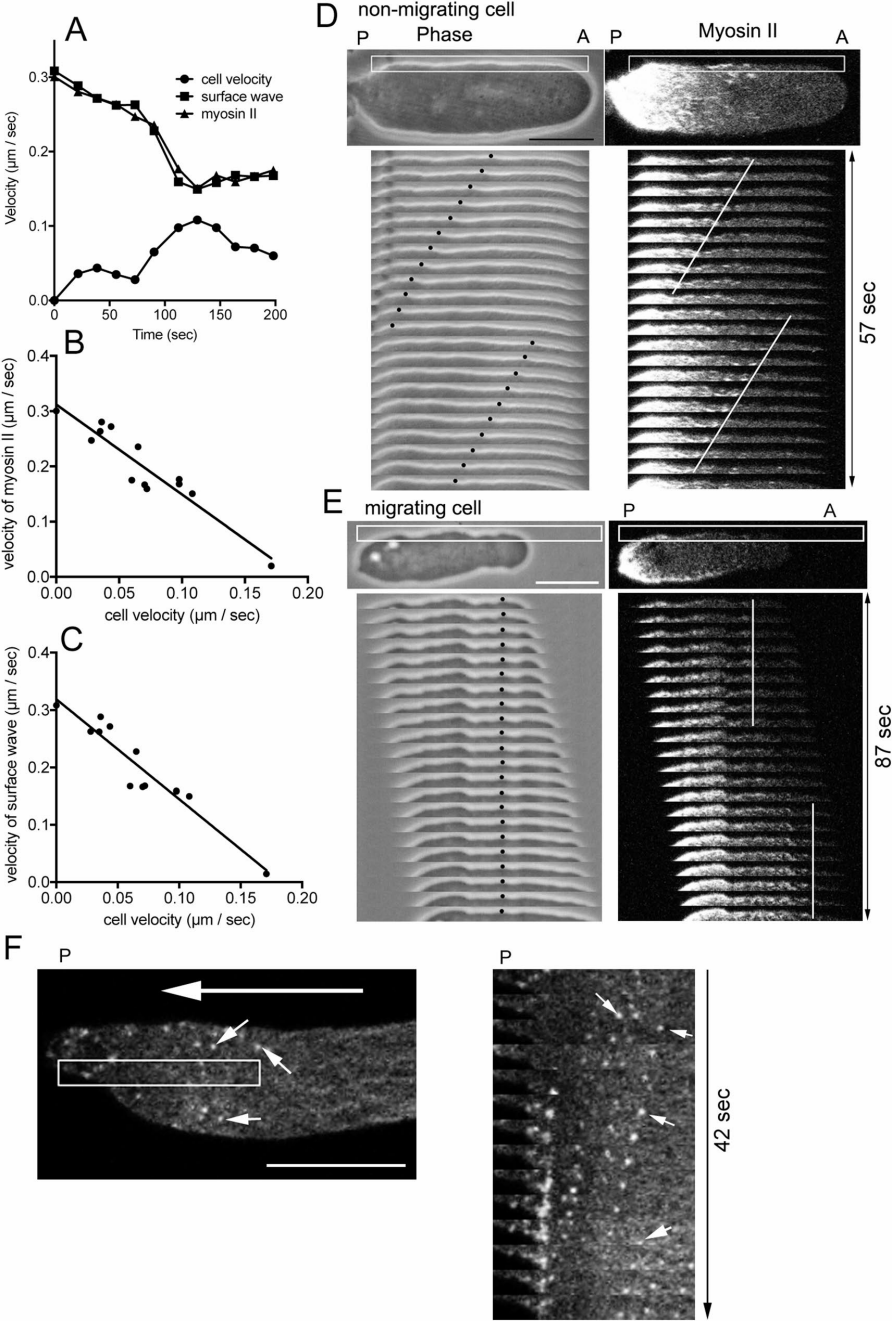
Velocity of the Flows is Inversely Proportional to the Cell Velocity (**A**) Temporal changes in flow velocities and cell velocity of a talin A/B null cell. As cell velocity increased, surface wave and myosin II velocities decreased. (**B**) Relationship between cell velocity and the velocity of the surface wave across multiple cells (*n* = 13). (**C**) Relationship between cell velocity and myosin II flow across multiple cells (*n* = 13). (**D** and **E**) Representative kymographs of surface wave and myosin II flow in non-migrating and migrating cells, respectively. Small “A” and “P” indicate anterior and posterior, respectively. Note that both the convex shape of the cell surface (black dots) and myosin II filaments (white lines) did not flow in the migrating cell (**E**) but did in the non-migrating cell (**D**). (**F**) A typical kymograph of flow of actin foci (small arrows) in a non-adherent cell expressing GFP-lifeact. Right panel shows a kymograph of the rectangle in left panel. Large white arrow indicates the flow direction. All microscopic images were taken by TIRF microscopy. Bars, 10 μm

As mentioned above, under agar-overlay conditions, cells initiated migration over time despite their inability to adhere to the substratum in such a confined space [5, 6, 29, 30]. Figure 3A illustrates representative temporal changes in cell velocity, surface wave velocity, and myosin II flow velocity in a single talin A/B null cell. Notably, an inverse relationship was observed—when cell migration velocity increased, the velocities of surface waves and myosin II flow decreased. Figure 3B and C quantitatively demonstrate this inverse proportionality across multiple cells.

Figures 3D and E present kymographs of surface wave and myosin II flow in non-migrating and migrating cells, respectively. In non-migrating cells (Fig. 3D), both surface waves and myosin II filaments exhibited retrograde flow. However, in rapidly migrating cells (Fig. 3E), these flows were undetectable.

We previously showed that *Dictyostelium* cells attach to substrate with multiple discrete attachment spots (actin foci), similar to focal adhesions [34] whereas they have neither canonical integrins nor well-defined extracellular matrix. Focal adhesion components such as vinculin and paxillin also exist in the spots [35, 36]. The actin foci did not move relative to the substrate in adherent condition and finally shed on the substrate after the cells move away [34]. Figure 3F shows retrograde flow of actin foci (small white arrows) in non-migrating talin A/B null cell [34]. It is plausible that nascent actin foci may not engage the clutch mechanism, allowing actomyosin flow to persist.

These findings suggest that the driving force underlying actomyosin flows and surface waves may be converted into cell migration, supporting the clutch hypothesis.

### Turnover of actin and myosin II filaments to sustain the flows

**Fig. 4:**
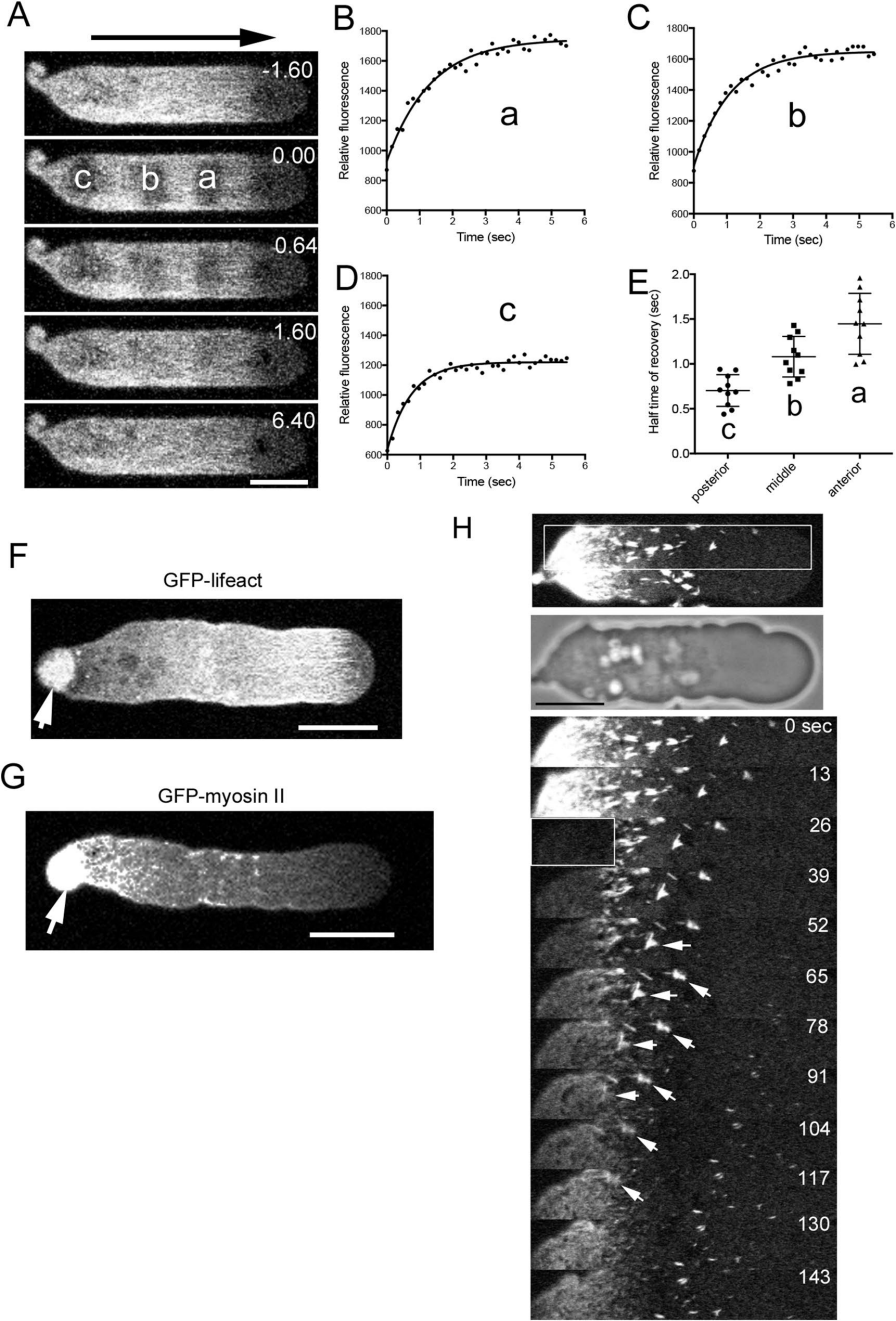
Turnover of Actin and Myosin II Filaments (**A**) A typical time course of fluorescence images of talin A/B null cells expressing GFP-lifeact after photobleaching. Fluorescence in three regions (anterior, middle, and posterior along the cell) was bleached (a, b, and c). Small “A” and “P” indicate anterior and posterior, respectively. (**B-D**) Time courses of fluorescence intensities at the bleached regions (a, b, and c). (**E**) Summary of half times of recovery. Data are presented as mean ± SD and analyzed by one-way ANOVA with Tukey’s multiple comparison test. ** *p* < 0.0001 (*n* = 10 for each). (F and G) Typical images of talin A/B null cells expressing GFP-lifeact (**F**) and GFP-myosin II (**G**) 15 min after applying 4 µM jasplakinolide. Arrows indicate aggregates at the posterior end. Similar results were obtained in at least 5 independent experiments. (**H**) Tracking of myosin II filaments after photobleaching the rear region. Upper two panels show confocal and phase-contrast images of a talin A/B null cell expressing GFP-myosin II. Lower panels show a kymograph of a white rectangle in the upper fluorescence image. When myosin II filaments reached around the posterior, they gradually decreased their fluorescence and finally disappeared (arrows). All microscopic images were taken by confocal microscopy. Bars, 10 μm

Despite the robust actin and myosin II retrograde flows, no excess accumulation of these filaments was observed at the cell posterior, suggesting that continuous turnover and recycling of their components are required. To investigate this, fluorescence recovery after photobleaching (FRAP) was performed in talin A/B null cells expressing GFP-Lifeact. Figure 4A illustrates a typical FRAP time course, where three small regions (a, b, and c) were simultaneously bleached. The fluorescence recovery kinetics for these areas are shown in Figs. 4B–D. Figure 4E summarizes the half-times across multiple experiments (*n* = 10 for each), revealing significantly faster actin turnover at the posterior region (c) compared to the anterior and middle regions.

To further assess the role of actin filament turnover, cells were treated with jasplakinolide, a membrane-permeable actin stabilizer. This treatment led to the substantial accumulation of actin filaments at the posterior (arrow in Fig. 4F), highlighting the necessity of rapid turnover for effective actin recycling. Notably, similar actin aggregates have been observed at the posterior of migrating adherent cells treated with jasplakinolide [18, 37]. Figure 4G shows that myosin II similarly accumulated at the posterior under jasplakinolide treatment.

Due to the high density of myosin II filaments at the posterior, individual filaments were difficult to track. To address this, the posterior region was photobleached, and individual myosin II filaments moving toward this area were monitored. As filaments reached the posterior, they progressively lost fluorescence and eventually disappeared (arrows in Fig. 4H), indicating their disassembly. Similar results were obtained in at least ten independent experiments. Taken together, these results demonstrate that actin and myosin II filaments preferentially disassemble at the posterior, facilitating their continuous recycling and sustaining cortical flows.

### Enhanced cortical flows in detached cells during cytokinesis

*Dictyostelium* cells do not detach from the substrate during cell division and divide using both the constriction of contractile ring and the traction force of daughter cells migrating in the opposite directions. Actin and myosin II have been reported to undergo equator-directed cortical flows during cytokinesis, contributing to contractile ring formation in various organisms [38, 39]. Previously, we demonstrated slow myosin II flow toward the equator in dividing *Dictyostelium* cells [19, 27].

**Fig. 5:**
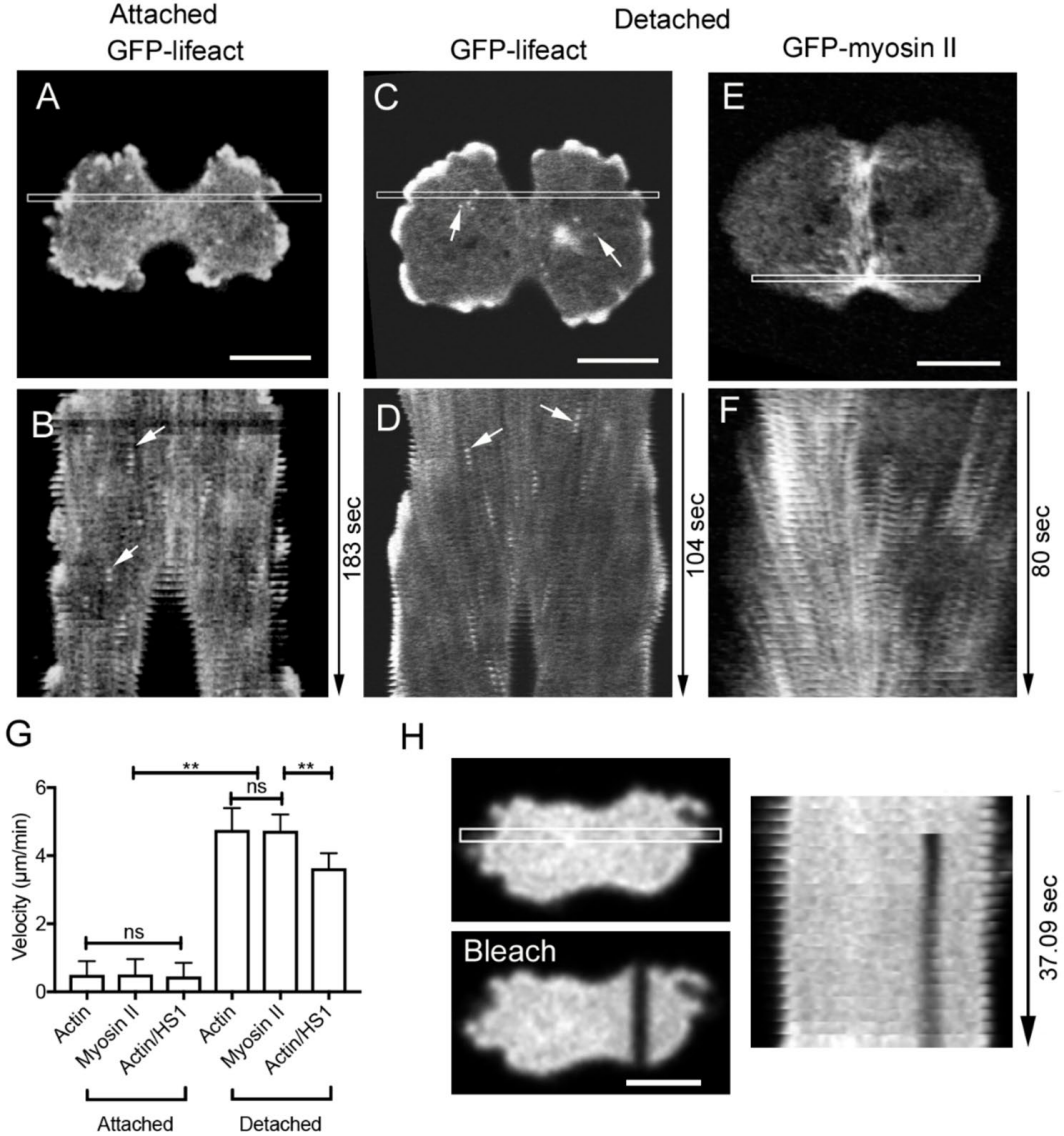
Cortical Flows are Enhanced by Detaching the Cell from the Substratum During Cytokinesis (**A-D**) A typical TIRF images and kymographs of dividing wild-type cells expressing GFP-lifeact on uncoated coverslip (**A** and **B**) and Lipidure-coated coverslip (**C** and **D**). The cells were mildly pressed with an agarose block to observe the ventral cortex by TIRF microscopy. Panel B and D show kymographs of the white rectangles of panel A and C, respectively. Arrows indicate actin foci. (**E** and **F**) A typical TIRF image and kymograph of dividing cells expressing GFP-myosin II. Panel F shows a kymograph of the white rectangle of panel E. (**G**) Summary of velocities of actin and myosin II flows in wild-type cells and myosin null cells (HS1) under adherent and non-adherent conditions, respectively. Data are presented as mean ± SD and analyzed by one-way ANOVA with Tukey’s multiple comparison test. ** *p* < 0.0001, ns, not significant, *P* > 0.05 (*n* = 10). (**H**) A typical photobleaching experiment of a dividing cell expressing GFP-cAR1 under confocal microscopy. Left two panels show fluorescence images before and after photobleaching, respectively. The right panel shows a kymograph in the white box in the right panel. Similar results were obtained in at least five independent experiments. Bars, 10 μm

Because both talin A/B null cells and EDTA-treated cells rarely underwent cytokinesis, we used Lipidure-coated coverslips to observe detached dividing cells. Figures 5A and B present a kymograph of actin dynamics in dividing cells on uncoated coverslip. Figures 5C-F present kymographs of actin and myosin II on Lipidure-coated coverslip. These data clearly show equator-directed actin and myosin II flows. Actin foci (white arrows) also exhibited equator-directed movement on non-adherent condition (D) whereas they remained almost stationary on adherent condition (B), suggesting that nascent actin foci do not engage the clutch mechanism.

Figure 5G summarizes the flow velocities under attached and detached conditions. In detached cells, actin and myosin II exhibited significantly faster equator-directed flow (4.76 ± 0.64 μm/min for actin and 4.73 ± 0.48 μm/min for myosin II, *n* = 10 each) compared to attached cells (0.50 ± 0.4 μm/min for actin and 0.51 ± 0.45 μm/min for myosin II, *n* = 10 each).

To assess the role of myosin II in driving actin flow, we examined actin velocity in myosin II-null cells, which averaged 3.63 ± 0.44 μm/min (*n* = 10). This velocity was significantly lower than that in wild-type cells, indicating that myosin II partially contributes to actin flow.

To determine whether membrane components undergo equator-directed flow, cells expressing GFP-cAR1 were subjected to partial photobleaching. Figure 5H shows that membrane components exhibited no net movement relative to the substratum.

*Dictyostelium* cells need traction force by the opposite migration of the daughter cells during cytokinesis in addition to the constriction of the contractile ring [6, 40]. Our findings suggest that the clutch hypothesis may explain cortical flow not only during cell migration but also during cytokinesis because the flows were observed upon detachment in both cases.

## Discussion

When detached from the substratum, *Dictyostelium* cells exhibited vigorous surface waves and retrograde actomyosin filament flows from the anterior to the posterior end of the cell. In animal cells, retrograde flow is typically limited to pseudopods or, at most, extends to the cell centroid. An exception is observed in the non-adherent subline of Walker 256 carcinosarcoma cells as far as we know, where flows extend to the rear end [5]. The region where retrograde flow occurs is likely determined by the site of clutch disengagement: if the clutch is disengaged at the anterior, the flow remains localized to that region.

Surface waves and actomyosin flows appear to be driven by the same underlying mechanism, as evidenced by their identical velocities. The primary driving force is attributed to actin polymerization at the anterior, as the flow velocity decreases toward the posterior, and inhibition of myosin II has minimal impact on flow velocity, whereas actin inhibition halts the flow. Myosin II filaments may be passively transported by actin flow, independent of their motor activity. This notion is supported by previous findings that motorless myosin II flows toward the cleavage furrow during cytokinesis [41].

The velocity of cortical flow is inversely proportional to cell migration velocity in detached *Dictyostelium* cells, akin to the inverse relationship between cell advance and retrograde flow in neuronal growth cones [7]. The interplay between focal adhesion, actin retrograde flow, and cell velocity has been demonstrated on substrates of varying adhesiveness [42–45]. The clutch hypothesis provides a rational explanation for these phenomena [9, 10, 46]. Our observation that actin foci were not fixed in position but instead flowed under detached conditions further supports the idea that the clutch is disengaged. Thus, the clutch hypothesis is well applicable to *Dictyostelium* cell migration.

Despite the robust flow of actin and myosin II filaments, they did not excessively accumulate at the posterior of the cell. FRAP experiments revealed that actin filaments turnover more rapidly in the posterior region than in the anterior or middle regions. Furthermore, stabilizing actin filaments with jasplakinolide resulted in their accumulation, forming a large aggregate at the rear of the cell. In the absence of the drug, actin filaments likely undergo disassembly at the posterior. Direct observation of individual myosin II filaments after photobleaching also suggested that myosin II disassembles at the rear of polarized cells. The assembly of myosin II is regulated by the phosphorylation of its heavy chains. We previously showed that myosin II mutant that cannot disassemble by replacing phosphorylatable threonines to alanine residues also form an aggregate at the posterior of migrating cells [18, 41]. These findings indicate that rapid filament turnover is essential for maintaining continuous cortical flow. Given that flow velocity varies with cell migration velocity, the turnover rate may be regulated by monitoring the accumulation of actomyosin at the posterior region.

The reason why actin retrograde flow velocity decreases toward the posterior remains unclear. One plausible explanation is that actin filaments assemble at the anterior and undergo transport, gradually dispersing toward the posterior. Alternatively, accelerated turnover at the posterior may enhance actin disassembly, which could in turn account for the apparent deceleration of the flow.

In this study, we discovered that actin and myosin II flows toward the equator during cytokinesis were significantly enhanced when cells were detached from the substratum. During cytokinesis, the two daughter cells exert traction forces at both poles, moving in opposite directions [40]. In the absence of cell-substratum adhesion (i.e., when the clutch is disengaged), this traction force may be converted into cortical actin and myosin II flows toward the equator, akin to the process observed in migrating cells.

This flow may contribute to contractile ring assembly by facilitating actomyosin accumulation at the equator. Two models have been proposed to explain actin accumulation in the contractile ring: the *de novo* synthesis model, which suggests that actin is newly polymerized at the cleavage furrow, and the cortical flow model, which posits that preexisting cortical actin is transported to the furrow [19, 38, 47]. When flow velocity was enhanced by detachment, actin did not accumulate significantly more at the furrow than in the attached condition. This finding challenges the cortical flow model; however, detachment may also accelerate actin disassembly, maintaining a steady actin level at the furrow. Notably, actin and myosin II turnover has been reported in contractile rings across various cell types [48–51]. In *Dictyostelium* cells, the half-life of actin and myosin II in the contractile ring is approximately 1 and 7 s, respectively [19, 52]. The actin turnover rate must be tightly regulated to maintain an appropriate actin concentration at the furrow. Therefore, we cannot exclude the cortical flow model, as the balance of actin assembly and disassembly is also critical for the *de novo* synthesis model. Further investigations are required to elucidate the molecular mechanisms governing actin homeostasis at the furrow.

Myosin II filaments disappeared as they approached the posterior of the cell, suggesting that filament disassembly occurs in this region. When three phosphorylatable threonine residues in the myosin II heavy chain were mutated to alanines (3ALA), mutant myosin II accumulated excessively at the posterior [18, 53], resembling the actin and myosin II aggregation observed in jasplakinolide-treated cells (Fig. 4F, G). Notably, myosin heavy chain kinase C, which promotes filament disassembly, is also localized at the posterior [54, 55].

Photobleaching experiments of cAR1 and tracking of attached beads did not reveal substantial membrane flow. This suggests that while the convex–concave shape of the cell surface propagates waves backward, membrane components themselves do not exhibit actual retrograde flow. Previous studies showed that beads attached to migrating cells remained stationary relative to the substratum under adherent conditions [18], and FRAP experiments demonstrated that membrane lipids do not undergo substantial movement relative to the substratum [33]. Beads may be physically linked to the cortical actin cytoskeleton via transmembrane proteins such as SibA, with a limited similarity to integrin ß [56], but this connection may be disrupted upon detachment.

## Conclusion

Detachment from the substratum induces cortical flow during both cell migration and cytokinesis in *Dictyostelium* cells. The clutch hypothesis provides a robust explanation for these phenomena. Although initially proposed to describe cell migration, this hypothesis also accounts for cortical flow during cytokinesis. Actin polymerization at the anterior serves as the primary driving force for retrograde flow, while myosin II filaments play a minor role. The dynamic turnover of actin and myosin II, involving continuous assembly and disassembly, enables sustained cortical flow. These findings provide new insights into the mechanisms governing retrograde flow in detached *Dictyostelium* cells, highlighting the critical role of cytoskeletal dynamics in both migration and cytokinesis. 

## Supplementary Information

Below is the link to the electronic supplementary material.

Supplementary Material 1: Supplementary movie S1: Retrograde flow of actin filaments. Cortical actin filaments show a retrograde flow upon detachment, which was observed by TIRF microscopy.

Supplementary Material 2: Supplementary movie S2: Retrograde flow of myosin II filaments. Cortical myosin II filaments show a retrograde flow upon detachment, which was observed by TIRF microscopy.

Supplementary Material 3: Supplementary movie S3: Retrograde flow of surface wave. Retrograde movement of convex-concave deformations on the cell surface was observed in detached cells by bright-field microscopy.

Supplementary Material 4: Supplementary movie S4: Effect of latrunculin A on the actin retrograde flow. Latrunculin A-treatment halted the flows of actin filaments and surface waves, and caused cells to adopt a more rounded morphology due to anterior retraction.

## Data Availability

The data are available from the corresponding author upon reasonable request.

## Abbreviations

cAR1: Cyclic adenosine monophosphate (cAMP) receptor 1
EDTA: Ethylenediamine tetraacetic acid
FRAP: Fluorescence recovery after photobleaching
GFP: Green fluorescent protein
TIRF: Total internal reflection fluorescence

## References

[1] Mitchison TJ, Cramer LP. Actin-based cell motility and cell locomotion. Cell. 1996;84:371–9. 10.1016/s0092-8674(00)81281-7. 8608590 10.1016/s0092-8674(00)81281-7

[2] Pollard TD, Borisy GG. Cellular motility driven by assembly and disassembly of actin filaments. Cell. 2003;112:453–65. 12600310 10.1016/s0092-8674(03)00120-x

[3] Friedl P, Entschladen F, Conrad C, Niggemann B, Zanker KS. CD4+ T lymphocytes migrating in three-dimensional collagen lattices lack focal adhesions and utilize beta1 integrin-independent strategies for polarization, interaction with collagen fibers and locomotion. Eur J Immunol. 1998;28(8):2331-2343. 10.1002/(sici)1521-4141(199808)28:08%3C2331::aid-immu2331%3E3.0.co;2-c. 10.1002/(SICI)1521-4141(199808)28:08<2331::AID-IMMU2331>3.0.CO;2-C 9710211

[4] Lämmermann T, Bader BL, Monkley SJ, Worbs T, Wedlich-Soldner R, Hirsch K, Keller M, Forster R, Critchley DR, Fassler R, Sixt M. Rapid leukocyte migration by integrin-independent flowing and squeezing. Nature. 2008;453:51–5. 10.1038/nature06887. 18451854 10.1038/nature06887

[5] Bergert M, Erzberger A, Desai RA, Aspalter IM, Oates AC, Charras G, Salbreux G, Paluch EK Force transmission during adhesion-independent migration. Nat Cell Biol. 2015;17:524–9. 10.1038/ncb3134. 25774834 10.1038/ncb3134 PMC6485532

[6] Taira R, Yumura S. A novel mode of cytokinesis without cell-substratum adhesion. Sci Rep. 2017;7:17694. 10.1038/s41598-017-17477-w. 29255156 10.1038/s41598-017-17477-w PMC5735089

[7] Lin CH, Forscher P. Growth cone advance is inversely proportional to retrograde F-actin flow. Neuron. 1995;14:763–71. 7536426 10.1016/0896-6273(95)90220-1

[8] Henson JH, Svitkina TM, Burns AR, Hughes HE, MacPartland KJ, Nazarian R, Borisy GG. Two components of actin-based retrograde flow in sea urchin coelomocytes. Mol Biol Cell. 1999;10:4075–90. 10588644 10.1091/mbc.10.12.4075 PMC25744

[9] Mitchison T, Kirschner M. Cytoskeletal dynamics and nerve growth. Neuron. 1988;1:761–72. 3078414 10.1016/0896-6273(88)90124-9

[10] Jay DG. The clutch hypothesis revisited: ascribing the roles of actin-associated proteins in filopodial protrusion in the nerve growth cone. J Neurobiol. 2000;44:114–25. 10934316 10.1002/1097-4695(200008)44:2<114::aid-neu3>3.0.co;2-8

[11] Yamashiro S, Watanabe N A new link between the retrograde actin flow and focal adhesions. J Biochem. 2014;156:239–48. 10.1093/jb/mvu053. 25190817 10.1093/jb/mvu053

[12] Cramer LP, Mitchison TJ. Investigation of the mechanism of Retraction of the cell margin and Rearward flow of nodules during mitotic cell rounding. Mol Biol Cell. 1997;8:109–19. 9017599 10.1091/mbc.8.1.109 PMC276063

[13] Suter DM, Errante LD, Belotserkovsky V, Forscher P. The Ig superfamily cell adhesion molecule, apCAM, mediates growth cone steering by substrate-cytoskeletal coupling. J Cell Biol. 1998;141:227–40. 9531561 10.1083/jcb.141.1.227 PMC2132711

[14] Hu K, Ji L, Applegate KT, Danuser G, Waterman-Storer CM. Differential transmission of actin motion within focal adhesions. Science. 2007;315:111–5. 17204653 10.1126/science.1135085

[15] Wang YL. Flux at focal adhesions: slippage clutch, mechanical gauge, or signal depot. Sci STKE. 2007;2007:pe10. 10.1126/stke.3772007pe10. 17356172 10.1126/stke.3772007pe10

[16] Lin CH, Espreafico EM, Mooseker MS, Forscher P. Myosin drives retrograde F-actin flow in neuronal growth cones. Neuron. 1996;16:769–82. 8607995 10.1016/s0896-6273(00)80097-5

[17] Svitkina TM, Verkhovsky AB, McQuade KM, Borisy GG. Analysis of the actin-myosin II system in fish epidermal keratocytes: mechanism of cell body translocation. J Cell Biol. 1997;139:397–415. 9334344 10.1083/jcb.139.2.397 PMC2139803

[18] Yumura S, Itoh G, Kikuta Y, Kikuchi T, Kitanishi-Yumura T, Tsujioka M. Cell-scale dynamic recycling and cortical flow of the actin-myosin cytoskeleton for rapid cell migration. Biol Open. 2013;2:200–9. 10.1242/bio.20122899. 23430058 10.1242/bio.20122899 PMC3575654

[19] Yumura S. Myosin II dynamics and cortical flow during contractile ring formation in *Dictyostelium* cells. J Cell Biol. 2001;154:137–46. 11448996 10.1083/jcb.200011013 PMC2196877

[20] Yumura S, Hashima S, Muranaka S. Myosin II does not contribute to wound repair in *Dictyostelium* cells. Biol Open. 2014;3:966–73. 10.1242/bio.20149712. 25238760 10.1242/bio.20149712 PMC4197445

[21] Pervin MS, Itoh G, Talukder MSU, Fujimoto K, Morimoto YV, Tanaka M, Ueda M, Yumura S. A study of wound repair in *Dictyostelium* cells by using novel laserporation. Sci Rep. 2018;8:7969. 10.1038/s41598-018-26337-0. 29789591 10.1038/s41598-018-26337-0 PMC5964096

[22] Talukder MSU, Pervin MS, Tanvir MIO, Fujimoto K, Tanaka M, Itoh G, Yumura S. Ca2+-calmodulin dependent wound repair in *Dictyostelium* cell membrane. Cells. 2020;9:1058. 10.3390/cells9041058. 32340342 10.3390/cells9041058 PMC7226253

[23] Yumura S, Matsuzaki R, Kitanishi-Yumura T. Introduction of macromolecules into living *Dictyostelium* cells by electroporation. Cell Struct Funct. 1995;20:185–90. 10.1247/csf.20.185. 7586008 10.1247/csf.20.185

[24] Yumura S. A novel low-power laser-mediated transfer of foreign molecules into cells. Sci Rep. 2016;6:22055. 10.1038/srep22055. 26902313 10.1038/srep22055 PMC4763237

[25] Yumura S, Mori H, Fukui Y. Localization of actin and myosin for the study of ameboid movement in *Dictyostelium* using improved Immunofluorescence. J Cell Biol. 1984;99:894–9. 6381508 10.1083/jcb.99.3.894 PMC2113401

[26] Fukui Y, Yumura S, Yumura TK, Mori H. Agar overlay method: high-resolution Immunofluorescence for the study of the contractile apparatus. Methods Enzymol. 1986;134:573–80. 10.1016/0076-6879(86)34122-3. 3547039 10.1016/0076-6879(86)34122-3

[27] Yumura S, Ueda M, Sako Y, Kitanishi-Yumura T, Yanagida T. Multiple mechanisms for accumulation of myosin II filaments at the equator during cytokinesis. Traffic. 2008;9:2089–99. 18939956 10.1111/j.1600-0854.2008.00837.x

[28] Grespin DB, Niven TG, Babson RO, Kushner EJ. Lipidure-based micropattern fabrication for stereotyping cell geometry. Sci Rep. 2023;13:20451. 10.1038/s41598-023-47516-8. 37993505 10.1038/s41598-023-47516-8 PMC10665372

[29] Malawista SE, de Boisfleury Chevance A, Boxer LA. Random locomotion and chemotaxis of human blood polymorphonuclear leukocytes from a patient with leukocyte adhesion deficiency-1: normal displacement in close quarters via chimneying. Cell Motil Cytoskeleton. 2000;46::183–9. 10.1002/1097-0169(200007)46:3%3C183::aid-cm3%3E3.0.co;2-2. 10913965 10.1002/1097-0169(200007)46:3<183::AID-CM3>3.0.CO;2-2

[30] Lämmermann T, Germain RN The multiple faces of leukocyte interstitial migration. Semin Immunopathol. 2014;36:227–51. 10.1007/s00281-014-0418-8. 24573488 10.1007/s00281-014-0418-8 PMC4118216

[31] Yumura S, Kitanishi-Yumura T. Immunoelectron microscopic studies of the ultrastructure of myosin filaments in *Dictyostelium discoideum*. Cell Struct Funct. 1990;15:343–54. 2128211 10.1247/csf.15.343

[32] Yumura S. Reorganization of actin and myosin II in *Dictyostelium* amoeba during stimulation by cAMP. Cell Struct Funct. 1993;18:379–88. 10.1247/csf.18.379. 8033219 10.1247/csf.18.379

[33] Tanaka M, Kikuchi T, Uno H, Okita K, Kitanishi-Yumura T, Yumura S. Turnover and flow of the cell membrane for cell migration. Sci Rep. 2017;7:12970. 10.1038/s41598-017-13438-5. 29021607 10.1038/s41598-017-13438-5 PMC5636814

[34] Uchida KS, Yumura S. Dynamics of novel feet of *Dictyostelium* cells during migration. J Cell Sci. 2004;117:1443–55. 15020673 10.1242/jcs.01015

[35] Bukharova T, Weijer G, Bosgraaf L, Dormann D, van Haastert PJ, Weijer CJ. Paxillin is required for cell-substrate adhesion, cell sorting and slug migration during *Dictyostelium* development. J Cell Sci. 2005;118:4295–310. 10.1242/jcs.02557. 16155255 10.1242/jcs.02557

[36] Nagasaki A, Kanada M, Uyeda TQ. Cell adhesion molecules regulate contractile ring-independent cytokinesis in *Dictyostelium discoideum*. Cell Res. 2009;19:236–46. 10.1038/cr.2008.318. 19065153 10.1038/cr.2008.318

[37] Lee E, Shelden EA, Knecht DA. Formation of F-actin aggregates in cells treated with actin stabilizing drugs. Cell Motil Cytoskeleton. 1998;39:122–33. 9484954 10.1002/(SICI)1097-0169(1998)39:2<122::AID-CM3>3.0.CO;2-8

[38] Cao LG, Wang YL. Mechanism of the formation of contractile ring in dividing cultured animal cells. II. Cortical movement of microinjected actin filaments. J Cell Biol. 1990;111:1905–11. 2229180 10.1083/jcb.111.5.1905 PMC2116328

[39] Reymann AC, Staniscia F, Erzberger A, Salbreux G, Grill SW. Cortical flow aligns actin filaments to form a furrow. Elife. 2016;5:e17807. 10.7554/eLife.17807 PMC5117871 27719759

[40] Jahan MGS, Yumura S. Traction force and its regulation during cytokinesis in *Dictyostelium* cells. Eur J Cell Biol. 2017;96:515–28. 10.1016/j.ejcb.2017.06.004. 28633918 10.1016/j.ejcb.2017.06.004

[41] Yumura S, Uyeda TQ. Myosin II can be localized to the cleavage furrow and to the posterior region of *Dictyostelium* amoebae without control by phosphorylation of myosin heavy and light chains. Cell Motil Cytoskeleton. 1997;36:313–22. 9096954 10.1002/(SICI)1097-0169(1997)36:4<313::AID-CM2>3.0.CO;2-6

[42] Gupton SL, Waterman-Storer CM. Spatiotemporal feedback between actomyosin and focal-adhesion systems optimizes rapid cell migration. Cell. 2006;125:1361–74. 10.1016/j.cell.2006.05.029. 16814721 10.1016/j.cell.2006.05.029

[43] Alexandrova AY, Arnold K, Schaub S, Vasiliev JM, Meister JJ, Bershadsky AD, Verkhovsky AB. Comparative dynamics of retrograde actin flow and focal adhesions: formation of nascent adhesions triggers transition from fast to slow flow. PLoS ONE. 2008;3:e3234. 18800171 10.1371/journal.pone.0003234 PMC2535565

[44] Barnhart EL, Lee KC, Keren K, Mogilner A, Theriot JA. An adhesion-dependent switch between mechanisms that determine motile cell shape. PLoS Biol. 2011;9:e1001059. 10.1371/journal.pbio.1001059. 21559321 10.1371/journal.pbio.1001059 PMC3086868

[45] Shao D, Levine H, Rappel WJ. Coupling actin flow, adhesion, and morphology in a computational cell motility model. Proc Natl Acad Sci U S A. 2012;109:6851–6. 10.1073/pnas.1203252109. 22493219 10.1073/pnas.1203252109 PMC3344950

[46] Renkawitz J, Schumann K, Weber M, Lammermann T, Pflicke H, Piel M, Polleux J, Spatz JP, Sixt M. Adaptive force transmission in amoeboid cell migration. Nat Cell Biol. 2009;11:1438–43. 10.1038/ncb1992. 19915557 10.1038/ncb1992

[47] Pollard TD. Nine unanswered questions about cytokinesis. J Cell Biol. 2017;216:3007–16. 10.1083/jcb.201612068. 28807993 10.1083/jcb.201612068 PMC5626534

[48] Pelham RJ, Chang F. Actin dynamics in the contractile ring during cytokinesis in fission yeast. Nature. 2002;419:82–6. 10.1038/nature00999. 12214236 10.1038/nature00999

[49] Murthy K, Wadsworth P. Myosin-II-dependent localization and dynamics of F-actin during cytokinesis. Curr Biol. 2005;15:724–31. 10.1016/j.cub.2005.02.055. 15854904 10.1016/j.cub.2005.02.055

[50] Kondo T, Hamao K, Kamijo K, Kimura H, Morita M, Takahashi M, Hosoya H. Enhancement of myosin II/actin turnover at the contractile ring induces slower furrowing in dividing HeLa cells. Biochem J. 2011;435:569–76. 10.1042/BJ20100837. 21231914 10.1042/BJ20100837

[51] Masud Rana AY, Tsujioka M, Miyagishima S, Ueda M, Yumura S Dynamin contributes to cytokinesis by stabilizing actin filaments in the contractile ring. Genes Cells. 2013;18:621–35. 10.1111/gtc.12060. 23679940 10.1111/gtc.12060

[52] Fujimoto K, Tanaka M, Rana AYKMM, Jahan MGS, Itoh G, Tsujioka M, Uyeda TQP, Miyagishima SY, Yumura S. Dynamin-like protein B of *Dictyostelium* contributes to cytokinesis cooperatively with other dynamins. Cells. 2019;8(8):781. 10.3390/cells8080781. 10.3390/cells8080781 PMC6721605 31357517

[53] Yumura S, Uyeda TQ. Transport of myosin II to the Equatorial region without its own motor activity in mitotic *Dictyostelium* cells. Mol Biol Cell. 1997;8:2089–99. 9348544 10.1091/mbc.8.10.2089 PMC25674

[54] Nagasaki A, Itoh G, Yumura S, Uyeda TQ. Novel myosin heavy chain kinase involved in disassembly of myosin II filaments and efficient cleavage in mitotic *Dictyostelium* cells. Mol Biol Cell. 2002;13:4333–42. 10.1091/mbc.e02-04-0228. 12475956 10.1091/mbc.E02-04-0228 PMC138637

[55] Yumura S, Yoshida M, Betapudi V, Licate LS, Iwadate Y, Nagasaki A, Uyeda TQ, Egelhoff TT. Multiple myosin II heavy chain kinases: roles in filament assembly control and proper cytokinesis in *Dictyostelium*. Mol Biol Cell. 2005;16:4256–66. 10.1091/mbc.e05-03-0219. 15987738 10.1091/mbc.E05-03-0219 PMC1196335

[56] Cornillon S, Froquet R, Cosson P Involvement of Sib proteins in the regulation of cellular adhesion in dictyostelium discoideum. Eukaryot Cell. 2008;7:1600–5. 10.1128/EC.00155-08. 18676957 10.1128/EC.00155-08 PMC2547077

